# Maternal health care service seeking behaviors and associated factors among women in rural Haramaya District, Eastern Ethiopia: a triangulated community-based cross-sectional study

**DOI:** 10.1186/s12978-016-0270-5

**Published:** 2017-01-13

**Authors:** Dereje Kifle, Telake Azale, Yalemzewod Assefa Gelaw, Yayehirad Alemu Melsew

**Affiliations:** 1 Department of Health Education, Institute of Public Health, College of Medicine and Health Science, University of Gondar, Gondar, Ethiopia; 2 Department of Epidemiology and Biostatistics, Institute of Public Health, College of Medicine and Health Science, University of Gondar, Gondar, Ethiopia

**Keywords:** Health care seeking, Antenatal care, Postnatal care, Delivery, Women, Northeast Ethiopia

## Abstract

**Background:**

Regular utilization of maternal health care services reduces maternal morbidity and mortality. This study assessed the maternal health care seeking behavior and associated factors of reproductive age women in rural villages of Haramaya district, East Ethiopia.

**Methods:**

Community based cross sectional study supplemented with qualitative data was conducted in Haramaya district from November 15 to Decemeber 30, 2015. A total of 561 women in reproductive age group and who gave birth in the last 2 years were randomly included. Bivariate and multivariate logistic regressions model was used to identify the associated factors. Odds ratios with 95% CI were used to measure the strength of association.

**Result:**

Maternal health care service seeking of women was found as; antenatal care 74.3% (95% CI; 72.5, 76.14), attending institutional delivery 28.7% (95% CI; 26.8, 30.6) and postnatal care 22.6% (95% CI; 20.84, 24.36). Knowledge of pregnancy complications, Educational status, and religion of women were found to be significantly associated with antenatal health care, delivery and postnatal health care service seeking behaviours triangulated with individual, institutional and socio-cultural qualitative data.

**Conclusion:**

The maternal health care service seeking behavior of women in the study area was low. Educational status of the women, birth order and knowledge about pregnancy complications were the major factors associated with maternal health care service seeking behavior Focused health education with kind and supportive health care provider counseling will improve the maternal health care seeking behaviors of women.

## Plain English summary

The health of women during their pregnancy and delivery is vital for the mother and their children. In Ethiopia, to keep the health and safety of mothers at all levels of the health care facilities, there are services to be provided during pregnancy, during labor and delivery, and after delivery. If mothers utilize these services regularly they can be saved from sickness and death. The health care service utilization of women in the study area was low. According to the study, 74.3% of mothers used the health services during their pregnancy. However, less than one third of mothers deliver in the health institutions. Similarly, only 22.6% of mothers got health care service after they gave birth. Formal education and educating women about the bad signs of pregnancy can increase service utilization. In opposite, as the number of births that a woman gave increases her usage of the maternal health care decreases. Therefore, it is mainly recommended that educational opportunities for women have to be improved and much attention has to be given to women with higher number of children.

## Background

Maternal health is the health of women during pregnancy, childbirth and the postpartum period and maternal health care services are antenatal care (ANC), delivery care and postnatal care (PNC) services [1]. Maternal health has been becoming a global concern because the lives of millions of women in reproductive age can be saved through maternal health care services. Despite efforts that have been made to strengthen maternal health care services, maternal mortality is still high in most of the developing countries [2]. Every day, approximately 800 women die from preventable causes related to pregnancy and childbirth and 99% of all maternal deaths occur in developing countries [3]. Though the causes of maternal deaths are numerous and vary from place to place depending on various factors, the major ones are hemorrhage (mainly postpartum hemorrhage), hypertension and sepsis [4–7]. The large number of maternal mortality, especially in developing countries has been due to low level of maternal health care seeking behaviour. The low proportion of antenatal care compounded by the extremely low skilled person attended delivery might be some of the major reasons for the high maternal mortality persisting during the last decade [8]. Maternal health is a major challenge in most developing countries, including Ethiopia. With a maternal mortality ratio of 673/100,000 and 19,000 maternal deaths annually, Ethiopia is a major contributor to the world-wide death toll of mothers [9]**.**

Maternal health care service has been among the most important interventions to decrease maternal morbidity and mortality. Because of this fact, Ethiopia has given a special consideration to it in the last two decades. Maternal health is among the six priority areas in the reproductive health strategy of the country. Studies conducted in Ethiopia shows the increment of women who are getting maternal health care services from time to time. However, the maternal health care seeking behaviour of women is still low. In Ethiopia, one explanation for poor health outcomes among women is the nonuse of modern health care service by a great proportion of women in the country [10]. Only 41, 16 and 13% of women in Ethiopia receive antenatal, delivery care from health professionals and postnatal care, respectively [11].

Different factors have been found to be related with the utilization of maternal health care services. Generally, the associated factors can be categorized as socio-economic and demographic factors such as; educational status of the mother [12], maternal age [13], occupation [14], mothers knowledge of danger signs, marital status, women’s autonomy, birth order, religion, sex of household head, household income, household size, husband’s educational status, accessibility factors [15] and factors related with women’s perceived quality of maternal health care services [16]. Mothers knowledge of danger signs [17–19] and autonomy [20] were reported as significant determinants of care utilization.

Inspite of the need and the efforts made to improve access to maternal health care services and reduce maternal mortality, maternal health-seeking behavior of women in Ethiopia has still been very limited especially among rural women. The efforts to improve women’s health are hampered due to poor maternal health-seeking behavior. Finding the reason behind this behavior is worth doing to design interventions for better utilization. As the maternal health care seeking behavior is a complex phenomenon which can be influenced by geo-cultural settings, it needs a contextual thorough investigation. More over, the inconsistency of findings of various researches in relation to the relationship between the maternal health care seeking behaviour and associated factors necessitates this research. Thus, the aim of this study was to assess the maternal health seeking behavior and its associated factors among rural women of reproductive age in Haramaya district, eastern Ethiopia.

## Methods

### Study design and setting

**Fig. 1:**
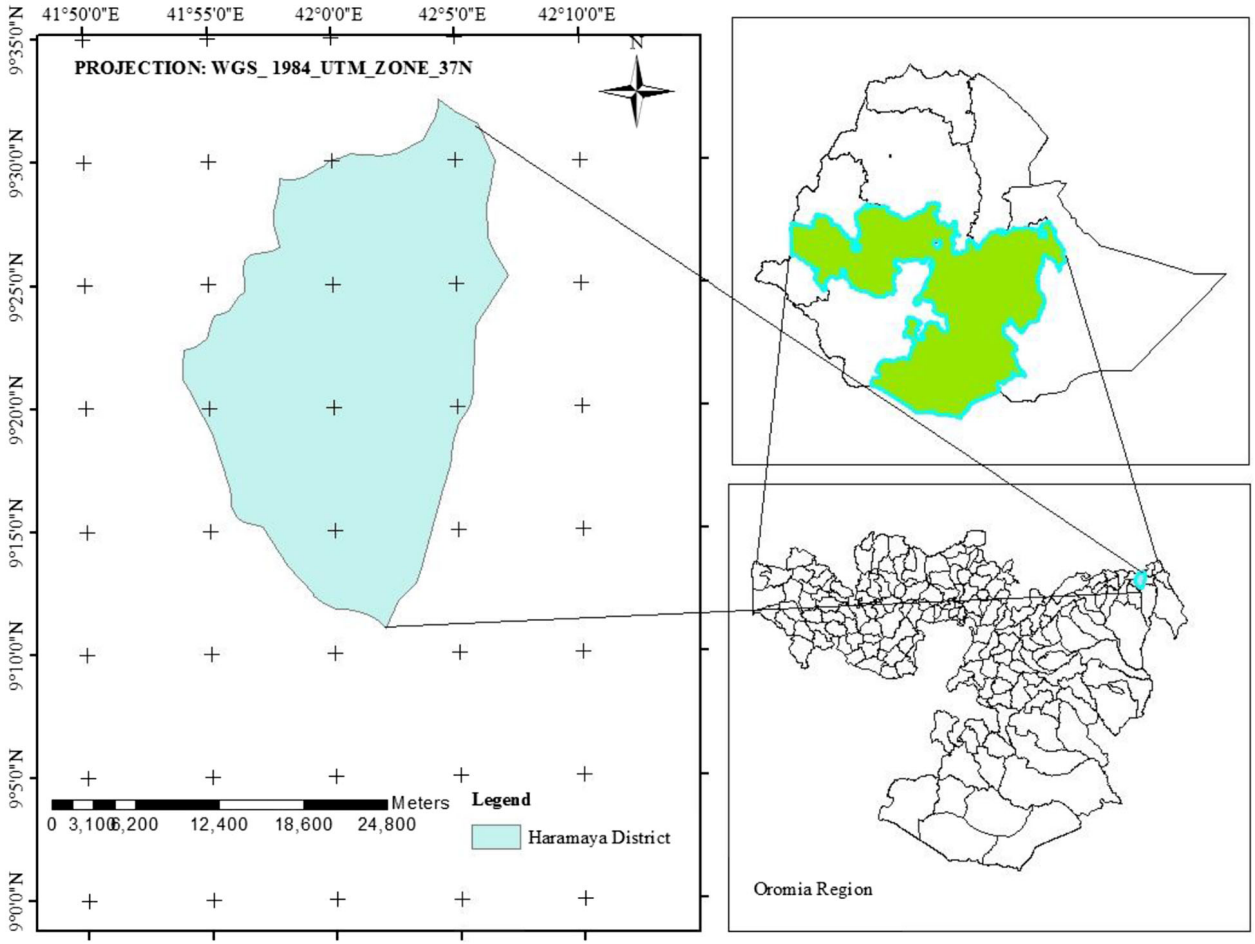
Rural districts of Haramaya, 2015

Community based cross sectional study supplemented with qualitative data was carried out from November 15 to Decemeber 30, 2015 in Haramaya district, Eastern Ethiopia. The study was conducted rural part of Haramaya district. The disrtict is found 505 km east of Addis Ababa, the capital of Ethiopia (Fig. 1). According to the information obtained from the district health bureau, the total population of the district was 271,394 of whom 138,376 were men and 133,018 were female. Among all the residents, 50,986 of them live in urban whereas the remaining 220,408 are residing in rural part of the district [21]. There were 34 Kebeles (*the smallest administrative units*) and of these 33 of the kebeles are rural. As to the health service facilities in the district; there were one district hospital, seven health centers, 34 health posts providing health care services.

### Study population and sampling

The study population included all women who gave birth in the last 2 years and residing in the rural villages of Haramaya district for at least 6 months.

The sample size was 561 women which were determined by single population proportion formula considering the multistage sampling technique. A two stage sampling technique was employed to select respondents for the study. First, eight rural kebeles were selected randomly from 33 rural kebeles. The number of women who gave birth in the last 2 years was obtained from health posts record in each kebele. Then, proportional to number of mothers who gave birth in the last 2 years, the respondents were chosen from each kebele using random sampling method for the quantitative data.

To supplement the quantitative data, FGD was conducted with three groups each consisting of 9–12 rural women and in-depth interview was done with ten maternal health care service providers.

Purposive sampling was used to select discussant women considering variability in age, time gave birth and social status. All women in FGD were not part of the quantitative study. Key informants for the in-depth interview were selected purposefully based on their involvement in maternal health care service provision.

### Variables and operational definition

The outcome variable was maternal health care seeking behaviour (antenatal, delivery and postnatal care). Whereas, Socio-economic and demographic related characteristics, access to maternal health care services, perceived quality of maternal health care services were taken as independent variables.

Maternal health**-** health of women during pregnancy, childbirth and the postpartum period.

Maternal death- death of a woman while pregnant or within 42 days of termination of pregnancy, irrespective of the duration and site of the pregnancy, from any cause related to or aggravated by the pregnancy or its management, but not from accidental or incidental causes*.*

Maternal morbidity- any injury, condition or symptom on women that resulted from or worsened by pregnancy.

Maternal health care seeking behaviour**-** utilization of maternal health care services (antenatal, delivery and postnatal care).

Antenatal care**-** the care received from healthcare professionals during pregnancy at least once.

Institutional delivery -delivery in public or private hospitals, clinics and health centers, attended by skilled attendants (midwifery, nurses, doctors, health officers).

Postnatal care**-** health care for the mother from immediately after the birth until around 6 weeks by health professionals.

### Data collection procedures

A structured and pretested questionnaire was used to collect the quantitative data via face to face interview technique. The questionnaire was adopted after reviewing different literature. The questionnaire was prepared in English and translated to the local language, Oromiffa, and then back to English by two different individuals to check the consistency. Twelve diploma holder nurses who were fluent in speaking Afan Oromo supervised by two BSc nurse and the investigator were involved in the data collection. Data collectors interview women at their households using structured questionnaires and interview guide. Focus group discussions were conducted at nearby village gathering areas with FGD checklist and tape recorder.

In order to ensure the quality of the data training was given to the data collectors and supervisors on basic skills, ways of obtaining consents and objectives of the study by the principal invigilator. Pretest was done for 5% of sample size in unselected *kebeles*. Definition of concepts and terms were made clear with a common language of the district to avoid ambiguity. The principal investigator did on-site supervision during the data collection period and review all filled questionnaires during the next morning of each data collection so as to identify incomplete and incoherent responses.

### Data processing and analysis

Each completed questionnaire was checked for completeness before data entry. Then the data were coded and entered in to a computer by using EPI Info version 7 and then data were exported to SPSS version 20 for analysis. Descriptive statistics were carried out to describe the study participants according to different characteristics and proportions were also computed. Binary logistic regression models were fitted to each; ANC, place of delivery and PNC to identify associated factors. Odds ratios with their 95% confidence interval (CI) were used to determine the strength and significance of association. *P* value less than 0.05 was considered as a level of significance. The qualitative data were analyzed with thematic analysis.

## Result

### Socio-economic and demographic characteristics of respondents

A total of 561 women who gave birth in the last 2 years of the study were interviewed. Of whom 96% were married. Two third (67.6%) of women were belonged to the age groups of 25–34 years. Majority, 91%, were Muslim religion followers and Oromo (94.5%) ethnic group. Regarding educational status 60.4% were unable to read and write. Four out of five and more (88.1%) of women were housewives. More than half, 58.6% has 1 to 4 household family size (Table 1).

**Table 1:** Socio-economic and demographic characteristics of women in Haramaya District, East Ethiopia, December 2015

Variables	Number	Percent
Age		
15–24	108	19.3
25–34	379	67.6
35 and above	74	13.2
Religion		
Christians (Orthodox/Protestant)	51	9.1
Muslim	510	90.9
Ethnicity		
Amhara	31	5.5
Oromo	530	94.5
Educational status		
Unable to read and write	339	60.4
Elementary	155	27.6
Secondary school and above	67	11.9
Occupation		
Housewife	494	88.1
Employee	41	7.3
Other (Petty trade/Student/Laboror)	26	4.6
Marital Status		
Currently not married	20	3.6
Married	541	96.4
Hosehold size		
1–4	329	58.6
5 and above	232	41.4
Educational level of the husband		
Unable to read and write	239	42.6
Elementary	225	40.1
Secondary school and above	97	17.3

### Maternal health care seeking behviour of women

The majority, 74.3% of women visited health facilities at least once for antenatal care. One fourth, 25.7% reported that they did not get ANC service throughout their pregnancy of the last children. Only one out of ten, 10% were had four antenatal care visits. Sixty nine percent of women reported that there were visited antenatal care clinics for the first time in second trimester of pregnancy. Less than one – third, 28.7% of women were attended institutional delivery with skilled health professionals. Moreover, 22% of women were utilized PNC service (Table 2).

**Table 2:** Utilization of ANC Service, institutional delivery and PNC by women in Haramaya District, East Ethiopia, December 2015

Variable	Number	Percent
At least one ANC		
No	144	25.7
Yes	417	74.3
Frequency of ANC		
None	144	25.7
1 time	69	12.3
2–3 times	290	51.7
4 or more times	58	10.3
Timing of the first ANC visit		
First Trimester	98	23.5
Second Trimester	288	69.1
Third Trimester	31	7.4
Gave birth at health facility		
No	400	71.3
Yes	161	28.7
PNC		
No	434	77.4
Yes	127	22.6

### Factors associated with antenatal care service utilization

Three different models were fitted to assess maternal health care service utilization. The firs model was fitted to assess associated factors of antenatal care service utilization. Variables such as educational status, occupational status, birth order of the last birth and knowledge about pregnancy danger signs were significantly associated with antenatal care service utilization. Women who able to read and write [AOR: 4.83, 95%CI: (2.06–11.33)] were 4.8 times more likely to seek antenatal care services than their counter part. Housewife women [AOR: 0.21, 95%CT: (0.06–0.71)] were 4.7 times more likely seek antenatal care service utilization when compared to women working petty trade and labour work. Women who gave more more(six and above) child were 89% less likely [AOR: 0.11, 95%CI: (0.03–0.43)] to seek ANC service as compared with who had one. Women who had knowledge on pregancy complication were by far utilized ANC services [AOR: 33.49, 95% CI: (14.56–77.02)] as compared to the counter parts (Table 3).

**Table 3:** Factors associated with antenatal care utilization among women who gave birth in the last 2 years in Haramaya District, East Ethiopia, December 2015

Characteristics	ANC service utilization		Crude OR (95% CI)	Adjusted OR (95% CI)
	Yes (%)	NO (%)		
Educational status				
Unable to read and write	217 (38.7)	122 (21.7)	1	1
Elementary/Read and write	138 (24.6	17 (3)	4.56 (2.66–7.91)	4.83 (2.06–11.33)*
Secondary school and above	62 (11.1)	5 (0.9)	6.97 (2.73–17.81)	5.68 (1.18–27.35)*
Occupation				
Housewife	361 (64.3)	133 (23.7)	1	1
employee (government/NGO)	37 (6.6)	4 (0.7)	3.41 (1.19–9.74)	1.17 (0.29–4.73)
Other (Petty trade/Student/Laboror)	19 (3.4	7 (1.2)	1 (0.41–2.43)	0.21 (0.06–0.71)*
Educational level of the husband				
Unable to read and write	147 (26.2)	92 (16.4)	1	1
Elementary/Read and write	184 (32.8)	41 (7.3)	2.81 (1.83–4.31)	1.28 (0.71–2.34)
Secondary school and above	86 (15.3)	11 (2)	4.89 (2.48–9.65)	0.59 (0.17–2.09)
Birth Order				
1	88 (15.7)	10 (1.8)	1	1
2–3	248 (44.2)	52 (9.3)	0.54 (0.26–1.11)	0.86 (0.35–2.09)
4–5	67 (11.9)	59 (10.5)	0.13 (0.06–0.27)	0.28 (0.10–0.81)*
6+	14 (2.5)	23 (4.1)	0.07 (0.03–0.18)	0.11 (0.03–0.43) *
Household size				
1–4	277 (49.4	52 (9.3	1	1
5 and above	140 (25	92 (16.4)	0.29 (0.19–0.43)	0.99 (0.51–1.90)
Knowledge about pregnancy complications				
No	143 (25.8)	137 (24.7)	1	1
Yes	268 (48.3)	7 (1.3)	33.49 (16.71–80.51)	33.49 (14.56–77.02)^*a^

^a^large odds ratio suggested that the small frequency in the category

*P value less than 0.05

### Factors associated with institutional delivery

The second model was fitted for institutional delivery. Accordingly, religion, educational status of the women, birth order, knowledge of pregnancy complications and ANC visit showed a significant association. Women Muslim religion followers were 80% less likely seek institutional delivery [AOR: 0.2, 95% CI: 0.08–.0.5)] as compared with their counter religion followers women. Women who had formal education and able to read and write were 2 times more likely to seek institutional delivery [AOR: 2.43 95%CI: 1.39–4.25] as compare to their counters. Women who gave their 4^th^ or 5^th^ births were 78% less likely to seek give birth at health faclity as compared to those who gave birth for the first time [AOR: 0.22, 95%CI: (0.08–0.57)]. Women who had a history of ANC visit [AOR: 3.63, 95%CI: (1.52–8.67)] and knowledge about pregnancy complications [AOR: 2.12, 95%CI: (1.26–3.56)] were more likely to seek institutional delivery as compared to their counterparts (Table 4).

**Table 4:** Factors associated with institutional delivery among women who gave birth in the last 2 years in Haramaya District, East Ethiopia, December 2015

Characteristics	Institutional delivery service utilization		Crude OR (95% CI)	Adjusted OR (95% CI)
	Yes (%)	NO (%)		
Religion				
Christians	42 (7.5)	9 (1.6)	1	1
Muslim	119 (21.2)	391 (69.7)	0.07 (0.03–0.14)	0.20 (0.08–0.50)*
Educational status				
Unable to read and write	47 (8.4)	292 (52)	1	1
Read and write/elementary	58 (10.3)	97 (17.3)	3.72 (2.37–5.82)	2.43 (1.39–4.25)*
Secondary school and above	56 (10)	11 (2)	31.63 (15.46–64.72)	12.95 (.35–38.56)*
Occupation				
Housewife	120 (21.4)	374 (66.7)	1	1
employee (government/NGO)	26 (4.6)	15 (2.7)	5.40 (2.77–10.54)	.43 (.13–1.135)
Other (Petty trade/Student/Laboror)	15 (2.7)	11 (2)	4.25 (1.90–9.50)	1.51 (.50–4.52)
Household size				
1–4	118 (21)	211 (37.6)	1	
5 and above	43 (7.7)	189 (33.7)	0.41 (0.27–0.61)	1.25 (0.66–2.34)
Educational level of the husband				
Unable to read and write	40 (7.1)	199 (35.5)	1	1
Read and write/elementary	58 (10.3)	167 (29.8)	5.40 (2.77–10.54)	0.83 (0.48–1.46)
Secondary school and above	63 (11.2)	34 (6.1)	4.25 (1.90–9.50)	1.18 (0.52–2.66)
Birth Order				
1	59 (10.5)	39 (7)	1	1
2–3	81 (14.4)	219 (39)	0.24 (0.15–0.39)	0.30 (0.17–0.54)*
4–5	16 (2.9)	110 (19.6)	0.96 (0.05–0.19	0.22 (0.08–0.57)*
6+	5 (0.9)	32 (5.7)	0.10 (0.04–0.29	0.37 (0.10–1.35)
Knowledge about pregnancy complications				
No	44 (7.9)	236 (42.5)	1	1
Yes	117 (21.1)	158 (28.5)	3.97 (2.66–5.93)	2.12 (1.26–3.56)*
Visit facility for ANC				
No	11 (2)	133 (23.7)	1	1
Yes	150 (26.7)	267 (47.6)	6.79 (5.59–12.97)	3.63 (1.52–8.67)*

*P value less than 0.05, Christian defined both Orthodox& Protestant regligion followers

### Factors associated with postnatal care utilization

The third model was fitted for postnatal care utilization. As a result, consistent with institutional delivery services Muslim religion follower women were 89% less likely to seek postnatal health care service than the counter parts [AOR: 0.11, 95%CI: (0.04–0.27)]. Women who had formal education attended and/or able to read and write husband [AOR: 4.0, 95%CT: (2.08–7.71)], a women with knowledge of pregnancy complications [AOR: 1.97, 05%CI: (1.08–3.61)] and a women who gave their last birth at health facility [AOR: 4.19, 95%CI: (2.38–7.37)] were more likely to seek postnatal cat = re service utilization as compared with their counterparts (Table 5).

**Table 5:** Factors associated with PNC among women who gave birth in the last 2 years in Haramaya District, East Ethiopia, December 2015

Characteristics	PNC service utilization		Crude OR (95% CI)	Adjusted OR (95% CI)
	Yes (%)	No (%)		
Religion				
Christians (Orthodox/Protestant)	42 (7.5)	9 (1.6)	1	1
Muslim	85 (15.2)	425 (75.8)	0.04 (0.20–0.09)	0.11 (0.04–0.27)*
Educational status				
Unable to read and write	44 (7.8)	295 (52.6)	1	1
Read and write/Elementary	37 (6.6)	118 (21)	2.10 (1.29–3.42)	0.94 (0.50–1.75)
Secondary school and above	46 (8.2)	21 (3.7)	14.69 (8.02–26.91)	2.24 (0.78–6.43)
Occupation				
Housewife	95 (16.9)	399 (71.1)	1	1
employee (government/NGO)	20 (3.6)	21 (3.7)	4.0 (2.08–7.68	0.54 (0.18–1.61)
Other (Petty trade/Student/Laboror)	12 (2.1)	14 (2.5)	3.6 (1.61–8.04	0.93 (0.28–3.07)
Household size				
1–4	92 (16.4)	237 (43.2)	1	1
5 and above	35 (6.2)	197 (35.1)	0.46 (0.30–0.71)	0.62 (0.31–1.26)
Educational level of the husband				
Unable to read and write	18 (3.2)	221 (39.4	1	
Read and write/Elementary	61 (10.9)	164 (29.2)	4.57 (2.51–2.60)	4 (2.08–7.71)*
Secondary school and above	48 (8.6)	49 (8.7)	12.03 (6.45–22.45)	3.38 (1.35–8.48)*
Birth Order				
1	32 (5.7)	66 (11.8)	1	1
2–3	73 (13)	227 (40.5)	0.66 (0.40–1.09)	1.76 (0.89–3.50)
4–5	20 (3.6)	106 (18.9)	0.39 (0.21–0.74)	3.09 (1.07–8.92)
6+	2 (0.4)	35 (6.2)	0.12 (.03–.52)	0.81 (0.13–5.20)
Knowledge about pregnancy complications				
No	35 (6.3)	245 (44.1)	1	1
Yes	92 (16.6)	183 (33)	3.52 (2.28–5.43)	1.97 (1.08–3.61)*
Visit facility for ANC				
No	13 (2.3)	131 (23.4)	1	1
Yes	114 (20.3)	303 (54)	3.79 (2.06–6.97)	1.27 (0.53–3.06)
Gave birth at health facility				
No	45 (8)	355 (63.3)	1	1
Yes	82 (14.6)	79 (14.1)	8.19 (5.29–12.69	4.19 (2.38–7.37)*

*P value less than 0.05

### Qualitative study result

We had three group discussion and in-depth interview for ten women who gave birth in the last 2 years of the interview period. The FGD and in-depth interview includes in three themes; individual, socio-cultural and institutional factors. In the following paragraph the participants’ maternal health care service explored as individual, socio-cultural and institutional factors.

#### Individual level

The most common individual level factor raised by the participant was being sick or feeling seek for maternal health care service utilization.

*“Though I know the importance of visiting health facilities, I do not do so unless I am feeling pain during pregnancy and after delivery.” Women, FGD*

This is also strengthen by the key informants (health service provider), the women in the study area felt that they [the women] would go to health facility only when they get sick.

Attitude and perception of the women towards the maternal health care services given in the health facilitywas also discussed as an individual level factor. It was indicated in the in-depth interview of health extension worker stated that

“*Women hesitated to come to health centers especially for delivery because of their fear that all women at health facility are giving birth in episiotomy (*cutting to widen the opening of the vagina) *which the women did not want. The women’s worry is because of what they heard when people are talking.”*

Moreover, the way how the women perceived the service at health facilities was also found individual level influencing factor for maternal health care service utilization. Discouraging woman by health care provider and long waiting time were raised as a contributor of individual level factors for not to seek maternal health care service utilization. FGD participants discussed that

“*There are some service providers that do not entertain customers properly. How do I go there if i do not get the services I need?”*

Reports from the in-depth interview also supported with health care service provider during FGD.

The way how women delivered in health facilities were also indicated as the third individual factor associated with maternal health care seeking behavior of women. As to the informant (midwife) women in the area were customarily giving birth by kneeling down. When they were told to sleep on the bed they always hesitatedbecause they did not feel at ease giving birth that way. A women also stated that,

*“I do not want to go to health facility for delivery because I do not feel comfortable sleeping on the delivery bed at the health center.”*

On the other hand, there were women who argued that they benefited from attending maternal health care services at health facilities and said that

*I got healthy child because I attended ANC and delivered at facility. I also received good care from the health service providers.*

#### Institutional level

Incomplete service due to unavailability of electric power, absence of some reagents, drugs and other important devices were found the main barriers across the health institutions in the study setting.

As to a midwife in a health center, when there was no electric power they found it difficult to give services especially delivery at night. While the newborn needs to be under heater, mostly we didn’t do keep the neonate in the heat because of regular electricity interruption. Related to this, the expectant women preferred to deliver at home than going to the health center.

A midwife mentioned that

“*It has been about four months that we finished reagents we used to test urine, blood.... because of this we could not give full maternal health care services that led women not to come to us.”*

Mostly, shortage of maternal health drugs is a notifiable chronic barriers across the country governmental health facilities. It was also mentioned by a woman in the FGD that they were facing problems to found the prescribed drugs in the health center pharmacy aside from the accessibility of health center and lack of convenient room and coaches for delivery services. A midwife indicated that as the rural women did not prefer to visit health facilities for maternal care service as they expected to visit due to transportation problem, scarcity of ambulance, poor road construction aside from familiarizations of food stuffs. Transportation problem is supported with a midwife since the district had only one ambulance for six health centers. A woman who participated in the FGD shared this view and said,

*“We wanted to deliver at health center/hospital but when we called the ambulance it came very late so that we were enforced to give birth at home.*” Women, FGD, Harmoia district

On the other hand, almost all women respondents from the study setting felt well by the cost of maternal health care services were provided free in all government health facilities.

#### Socio-cultural level

Most of the women in the study area were Muslims and respondents in the district were reported their religion influenced their maternal health care seeking behavior. An interview held with a HEW revealed that most Muslim women did not want to be attended by male birth attendants because of their religion. As such, health extension workers (health care providers in the district) revealed that religion existed the barrier at the level of the socio-cultural theme.

*“The Muslim women feel that they can only be touched and seen by their husbands. That is why they do not want to be attended by male midwives.” Health extension worker, In-depth interview, Haromia rural district*

A woman in the FGD argued that,

“Our *religion does not allow our naked body to be seen by any other person other than husbands. However, we are not sure whom we will be attended in the health facility. Even, we do not want to be uncovered so that our private parts do not be seen.”*

Despite unsatisfactory maternal health care service utilization in the district, postnatal care service was overlooked in the rural women of the study area. Discussant associated postnatal care service with follow-up of those who delivered unhealthy child. On the other hand, almost all women mentioned that they are drawn to attending PNC service at health facility because it was not customary in the study setting.

## Discussion

This study anticipated poor maternal health care service utilization in the rural settings and has demonstrated that societal, individual, sociodemographic and health service factors affect maternal health care seeking behaviour of women.

As to the findings of this study, the number of women who visited health facilities at least once during their recent pregnancy for antenatal care was 74.3% (95% CI:72.46,76.15) and only 10% (95% CI:8.7%,11.3%) of the women visited health facilities at least 4 times for antenatal care service utilization. This study is consistent with studies study done in East Wollega [22] and Southern Ethiopia [23]. It is, however, comparing to other countries the ANC utilization in the study area was low. Nearly 80% of respondents received ANC more than four times in Indonesia [24] and a qualitative study done in three Africa countries; Kenya (92%), Malawi (98%) and Ghana (92%) women received ANC services at least once [25]. In regard to the timing of the first ANC visit, our result shown that more than two third (69.1%) of women went to health center for ANC in the second trimester of their pregnancy whereas, less than one fourth (24%) of them visited health facility during the first trimester. This was also consistent with previous study done in Metekel Zone of Northwest Ethiopia [26]. The qualitative data also support this findings. Focused health education and counselling needs to be done using health extension worker and community health workers.

The current study reported that participants who had attend institutional delivery were very low. The study result is consistent with similar studies done in Ethiopia [27].

The result showed postnatal health care service in the study setting was very low. This was also consistent with study done in Dembecha district, Northwest Ethiopia [28]. This could be explained in to three reasons; (i) low institutional delivery in the study setting, (ii) low antenatal health coverage and (iii) lack of knowledge on the importance of the service.

This study found that the women educational status contributed to their antenatal health care service seeking behaviour in maternal health care service. Women able to read and write and formal education attended women were more likely to seek antenatal health care [16, 25, 28], and institutional delivery [11, 26, 29]. The finding of this study also suggested that women who had able to read write and formal education attended husband were more likely seek postnatal care service [13, 17, 30] which is in line with previous studies done in Ethiopia. It is understood that education is likely to enhance women autonomy and they are near to information and would have good knowledge [31].

Women who had a job (peaty trading) were less likely to seek antenatal health care service utilization than who had no formal jobs. This association might be explained as the timeconstraints. The petty traders and day laborers didnot earn for their livelihood unless they engaged in their daily activities so that they could not have time for ANC visit.

Birth order was found significantly associated with maternal health seeking behaviour of women, especially antenatal care and delivery service. It was investigated that utilization of antenatal care and delivery service declined with the increase in number of births. Given women had six and above safe delivery history, she may feel experience healthy and less likely seek maternal health care services [32]. Contrarily, when the number pregnancies she might feel as a risk mother and seek maternal health care services. The current study found that increasing birth order negatively associated with antenatal care service seeking.

The current study reported that knowledge of pregnancy complications was found a significant factors associated with maternal health care seeking behaviours in the study settings Likewise, other previous studies [11, 20] [29] showed women who had knowledge of pregnancy complications were by far more likely seek antenatal care, postnatal care and delivery service utilization than their counters.

The findings of this study also suggested that religion is associated with maternal health care service seeking behaviours of women. Muslim religion followers were found less likely to seek maternal health care seeking service as compared with Christian followers. This finding is consistent with studies done in Bangladesh [33]. The possible explanation could be Muslim women in the study area believed that their naked body could only be seen by their husband. This is also demonstrated with qualitative data in which religion was an influencing socio-cultural factors of maternal health care service seeking behaviour. They prefer female traditional birth attendant than skilled health care provider.

Additionally, place of birth was also shown significantly associated with seeking postnatal health service. Consistent with studies done in northwest Ethiopia [27], delivering at health facility led women to seek for PNC services.

Our study also showed that individual attitude towards health care providers and perceptions on the quality services provided in health facilities were mentioned as influencing factors for maternal health service seeking behaviours of women consistent with previous studies [8, 34].

Shortage of some medical stuffs, electricity interruptions, long waiting time, inaccessibility of transportation and distance of health facilities were frequently demonstrated in the qualitative data as institutional influencing factors of maternal health care service seeking behaviors and also supported with Tanzania study [35].

The potential limitations of this study were that the study may have been prone to recall bias, limited to rural resident women, didn’t consider the role of husband contribution for maternal health service and the tool did not include variables related to women’s experience of abortion, still birth and planned/unplanned pregnancies.

## Conclusion

Overall, maternal health care service utilization was found low in the study setting. Knowledge of pregnancy complications, Educational status, and religion of women were found to be significantly associated with antenatal health care, delivery and postnatal health care service seeking behaviours triangulated with individual, institutional and socio-cultural qualitative data.

## Abbreviations

ANC: Antenatal care
AOR: Adjusted odds ratio
COR: Crude odds ratio
DHS: Demographic and health survey
FGD: Focus group discusion
HEW: Health extension worker
PNC: Postnatal care
TBA: Traditional birth attendant
WHO: World Health Organization
